# Large scale analysis of protein conformational transitions from aqueous to non-aqueous media

**DOI:** 10.1186/s12859-018-2044-2

**Published:** 2018-01-30

**Authors:** Ana Julia Velez Rueda, Alexander Miguel Monzon, Sebastián M. Ardanaz, Luis E. Iglesias, Gustavo Parisi

**Affiliations:** 1 Departamento de Ciencia y Tecnología, CONICET, Universidad Nacional de Quilmes, Roque Sáenz Peña 352, B1876BXD Bernal, Provincia de Buenos Aires Argentina; 2 Laboratorio de Biocatálisis y Biotransformaciones, Departamento de Ciencia y Tecnología, Universidad Nacional de Quilmes, Roque Sáenz Peña 352, B1876BXD Bernal, Provincia de Buenos Aires Argentina

**Keywords:** Organic solvents, Conformational diversity, Biocatalysis, Protein dynamics

## Abstract

**Background:**

Biocatalysis in organic solvents is nowadays a common practice with a large potential in Biotechnology. Several studies report that proteins which are co-crystallized or soaked in organic solvents preserve their fold integrity showing almost identical arrangements when compared to their aqueous forms. However, it is well established that the catalytic activity of proteins in organic solvents is much lower than in water. In order to explain this diminished activity and to further characterize the behaviour of proteins in non-aqueous environments, we performed a large-scale analysis (1737 proteins) of the conformational diversity of proteins crystallized in aqueous and co-crystallized or soaked in non-aqueous media.

**Results:**

Using proteins’ experimentally determined conformational diversity taken from CoDNaS database, we found that proteins in non-aqueous media display much lower conformational diversity when compared to the corresponding conformers obtained in water. When conformational diversity is compared between conformers obtained in different non-aqueous media, their structural differences are larger and mostly independent of the presence of cognate ligands. We also found that conformers corresponding to non-aqueous media have larger but less flexible cavities, lower number of disordered regions and lower active-site residue mobility.

**Conclusions:**

Our results show that non-aqueous media conformers have specific structural features and that they do not adopt extreme conformations found in aqueous media. This makes them clearly different from their corresponding aqueous conformers.

**Electronic supplementary material:**

The online version of this article (10.1186/s12859-018-2044-2) contains supplementary material, which is available to authorized users.

## Background

Biocatalysis in organic solvents is nowadays a common practice with a large potential [1]. Basically, the use of organic solvents in enzyme catalysis offers several advantages over the use of an aqueous medium: it increases the solubility of many organic substrates and reagents, and decreases unwanted side reactions in water, it also enables enzyme separation at the end of the reaction and an easier purification of the reaction mixture due to enzyme insolubility in organic solvents and lower boiling points of common organic solvents [2]. Multiple studies suggest that protein environment influences their folding and thus their biological activity. The presence of ligands, ion concentration, temperature, the amount of bound water molecules and the presence of organic molecules such as solvent affect protein folding and protein structure [3]. Contrary to what may be believed in Biochemistry, as most enzymes evolved and performed their function in aqueous medium, several research studies have found that proteins co-crystallized or soaked in organic solvents preserve the integrity of the protein fold [4]. Several protein structures have been obtained in different organic solvents: chymotrypsin in hexane [5], subtilisin in anhydrous acetonitrile [6], trypsin in cyclohexane [7], egg-white lysozyme in the presence of alcohols [8] and thermolysin in isopropanol [9], just to mention some examples. The “kinetic trapping” theory explains that proteins in non-aqueous media remain in their native structure due to an increased amount of hydrogen-bonding between protein atoms resulting in a higher kinetic barrier for structural rearrangements [10]. This effect is related with the dehydration and resuspension that take place during crystallization [10–12]. It is accepted that solid lyophilized proteins have a different behaviour depending on the pH of the aqueous solution from which they were freeze-dried, remaining in the same conformation when transferred to a non-aqueous environment. In spite of this ‘structural conservation’, which is described in several research articles, it is well established that the catalytic activity of proteins in organic medium is lower than in water [13, 14]. Nevertheless, protein conformational transitions from aqueous to non-aqueous media as a possible cause of the observed lower activity in organic media is still under study. Even if most proteins co-crystallized or soaked in organic medium have the same structure as when they are obtained in a water medium, the preservation of the structure does not guarantee the same protein activity. For example, enzymes from thermophilic organisms are inactive at low temperatures due to a shortage of thermal energy, necessary to surmount the excess of rigidity that these proteins show [15]. Protein fold is conserved in its “native” state at low temperatures; however, the lack of dynamic features or conformational changes leads to inactivation. Hence, the term “native state” should comprise both structural and dynamical features of proteins. In this sense, it is well established that the native state is better understood as an ensemble of multiple structural conformers that coexist in equilibrium [16]. A wide range of structural differences among conformers have been explored in order to explain protein functions, from large relative domain movements [17], secondary and tertiary element rearrangements [18] and loop movements [19], to protein regions lacking a well-defined structure, which are known as intrinsically disordered proteins (IDPs) or intrinsically disordered regions (IDRs) [20].

Besides such large structural rearrangements, small movements are also observed for biological function and for catalysis [21, 22]. In a study of conformational changes in 60 enzymes between their apo and substrate-bound forms in aqueous solvents, Gutteridge and Thornton [23] reported that the motions of enzymes to binding their substrates were very small, and that enzymes requiring large motions represented a minor proportion. 75% of their data showed a C-alpha Root Mean Square Deviation (RMSD) of less than 1 Å, and 91% had an RMSD less than 2 Å with an average of 0.7 Å. Interestingly, they also noted that comparisons of apo structures for the same protein showed a RMSD of 0.5 Å, a value slightly below the observed apo and substrate-bound average. This observation was supported by the finding that small changes between conformers could still greatly affect catalytic parameters and thus, enzymes behaviour [22]. Moreover, in the last years several studies have revealed the importance of structures such as pockets, cavities and tunnels in protein function [24]. Briefly, these structures participate in the channeling of substrates and other ligands (cofactors, products, etc.) from the protein surface to the inner cavities which are probably associated with active or binding sites. The opening and closing of these structures through slight movements of very few residues (gatekeepers or bottleneck effect) could define active or inactive conformers [25].

In this research study, we have examined the structural changes observed in the transitions from aqueous to non-aqueous media in order to study conformational changes associated to these transitions, which could account for a lower enzymatic activity. The studies were carried out on sets of structures derived from the same protein. One group of these structures resulted from the crystallization process in aqueous media and another resulted from co-crystallization or soaking in non-aqueous media. Both kinds of structures were retrieved from CoDNaS (Conformational Diversity of the Native State) database [26]. We found the characteristic rigidity of proteins in the non-aqueous media already reported, which was evidenced by a low conformational diversity, along with a minor proportion of disorder regions which could reflect an overall lower protein flexibility. Furthermore, the extension of conformational diversity in aqueous media was not observed in the organic media, challenging the kinetic trapping hypothesis observations. Indeed, our results support the notion that conformers in non-aqueous media have unique features, which make them different from their corresponding conformers in aqueous media. The transitions in this environment seem to be characterized by minor changes in the exposed surface, higher ordered segments and cavities, and less conformational diversity.

## Results

### Comparison between aqueous and non-aqueous conformational diversity

**Fig. 1:**
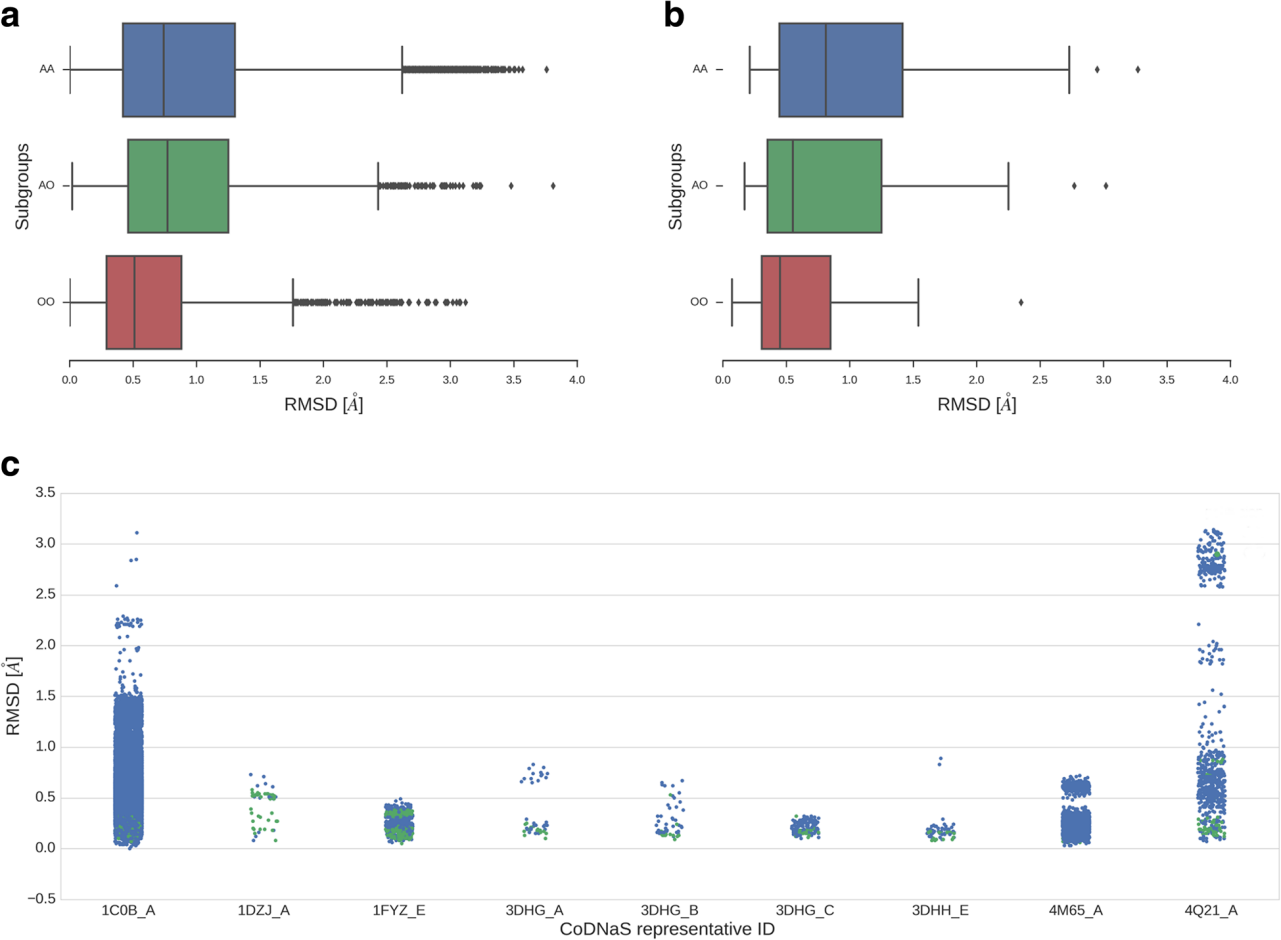
**a** Distributions of conformational diversity for the “large” and the “control” datasets. Distributions of conformational diversity for the “large” dataset measured in RMSD (Å) for the different subgroups AA (transitions from aqueous to aqueous environments in blue), AO (transitions from aqueous to non-aqueous environments in green) and OO (the transition from non-aqueous to non-aqueous environments in red). It is possible to observe that OO pairs have a much lesser conformational diversity than subgroup AA pairs. RMSD averages were 0.97, 0.94 and 0.68 Å for AA, AO and OO, respectively (observed medians: 0.74, 0.77 and 0.51 Å, respectively). *P*-values for comparisons between OO and AO and OO and AA were << 0.001, while AO and AA distributions showed no significant differences. **b** The “control” dataset shows the same behaviour than the “large” dataset (1A). **c** Distributions of conformational diversity for the most populated proteins of the “control” dataset taking into account all the conformer pairs. Distributions of conformational diversity of a representative pool of proteins from the “control” dataset measured in RMSD (Å) for the AA and OO subgroups. Transitions from aqueous to aqueous (AA) environments shown in blue and the transition from non-aqueous to non-aqueous (OO) environments are shown in green

In order to study the conformational diversity of proteins transitioning from non-aqueous to aqueous environments, we created two protein datasets with experimentally determined conformational diversity extracted from the CoDNaS database [26]. The control dataset results from a web scraping method followed by hand-curation for the collection of structures related with soaking and co-crystallization methods using organic solvents. The second dataset, which we called ‘large’, resulted from the text mining on the PDB (Protein Data Bank) files gathered using a list of frequently non-aqueous media used in crystallization process for the X-ray diffraction determination (see Methods). The resulting datasets include CoDNaS entries that possess at least two protein structures in non-aqueous environments and at least two other structures obtained in aqueous media, all of them for the same sequence (100% global sequence identity). Different structures of the same protein were taken as different conformers, which in CoDNaS are structurally compared using RMSD. Also, since one of the major factors influencing the extent of conformational diversity is the presence of ligands [27], and in order to focus our analysis in the structural changes due to medium transitions, we also selected pairs of conformers in their unbound forms as well as in their bound form. We finally obtained a total number of 1737 protein with conformers in both media (aqueous and non-aqueous) for the large dataset, and 33 proteins with conformers in both media for the control dataset. The tendencies found in both datasets were contrasted. Fig. 1 shows the distributions for the maximum RMSD pairs of proteins crystallized in different environments for both control and large datasets. The conformational changes observed in the transitions aqueous-aqueous and aqueous-non-aqueous environments (subgroups AA and AO, respectively) were statistically higher than the changes observed for the transition non-aqueous - non-aqueous (OO) (*P*-values for comparisons between OO and AO and OO and AA were << 0.001 while AO and AA distributions showed no significant differences). Interestingly, the RMSD of the maximum pairs distributions showed the same behaviour in both datasets: RMSD average 0.96, 0.97 and 0.71 Å for the large dataset, and 1.41, 1.02 and 0.50 Å for the control dataset (Fig. 1a and b, respectively). When we take into account all the conformer pairs—not only maximum pairs— for the control dataset for example, we can observe that proteins in non-aqueous media don’t seem to explore all the conformational space that they explore in water; non-aqueous conformer RMSD distributions are clearly restricted to a region around 0.5 Å, which is compatible with the crystallographic error [28] (Fig. 1c). It is important to note that these RMSD distributions are not influenced by fold/superfamily since fold classification and analysis of subpopulations showing large and small RMSD in the AA, AO and OO groups show no differential enrichment.

We also explored how the OO distributions could be affected by the presence of different organic solvents. In this sense, the OO distribution could be split into two distributions depending on how the conformers were obtained in different non-aqueous media. When OO conformers differ in the crystallization medium used, the average RMSD is 0.82 Å, while the RMSD is 0.63 Å when they differ in other conditions (for example, presence of post-translational modifications, differences in the oligomeric state), which shows the great influence that medium can have.

To gain further understanding of these structural changes, we analyzed, in the large dataset, differences in the secondary structure elements between conformers showing maximum RMSD. The average RMSD per site estimated for loops, alpha helices and beta sheets is shown in Table 1. We observed that the maximum variation for all the secondary elements is found in the AO pairs of conformers. As expected, the maximum value corresponded to the variation in the loops due to its intrinsic flexibility. Interestingly, the variation in AO pairs was above the average of the structural variation in the AA pairs, possibly reflecting that conformers in non-aqueous environments show some unique structural features when compared to the corresponding conformers in water. We also studied percentages of transitions between secondary structural elements, but no significant changes in the three different subgroups were found.

**Table 1:** Average RMSD of secondary structural elements between subgroups AA, AO and OO

Subgroup	AA [Å]	AO [Å]	OO [Å]
Alpha helix	0.78	0.91	0.62
Beta sheet	0.64	0.75	0.52
Loop	0.93	1.03	0.75

**Fig. 2:**
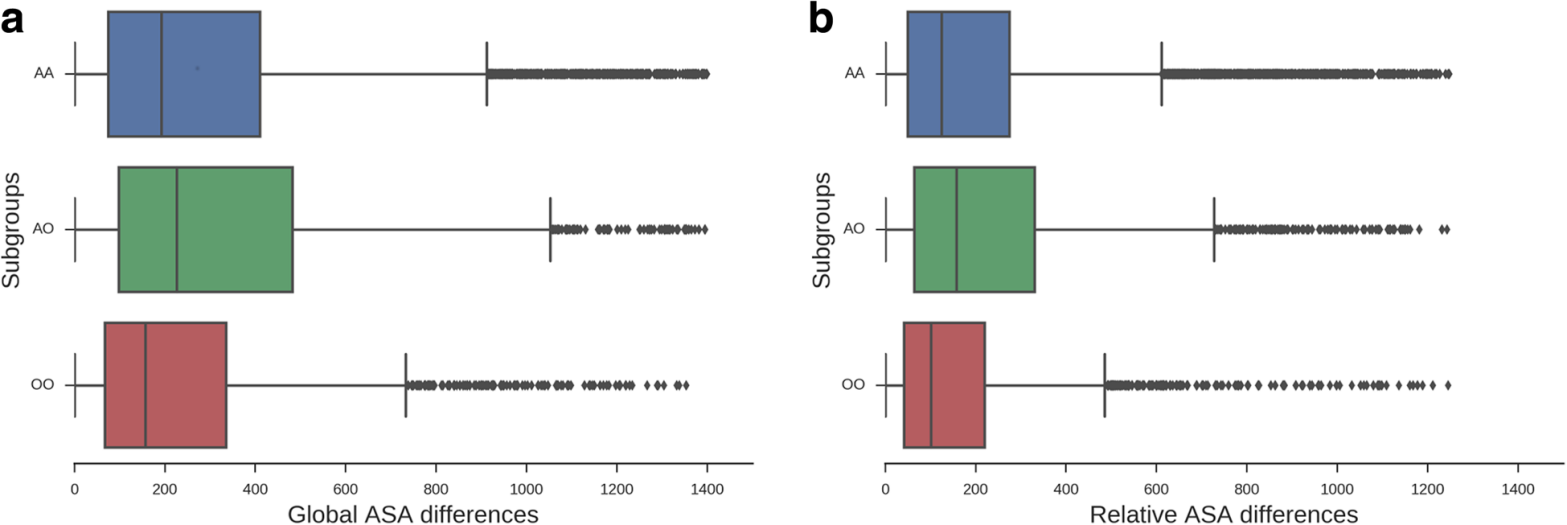
**a** Differences in the global ASA for the different subgroups of transitions AA (aqueous-aqueous environments in blue), AO (from aqueous to non-aqueous environments in green) and OO (from non-aqueous to non-aqueous environments in red). Subgroup AO shows the maximum differences evidencing bigger changes between conformers obtained in different solvents. Global ASA average differences were 412.78, 448.66 and 310.76 for AA, AO and OO, respectively (observed medians: 237.67, 257.70 and 162.87, respectively). *P*-values for global ASA differences comparisons were in all cases < < 0.001. **b** Differences in the relative ASA for the different transition subgroups AA (aqueous-aqueous environments in blue), AO (from aqueous to non-aqueous environments in green) and OO (the transition from non-aqueous to non-aqueous environments in red). Subgroup AO shows the maximum differences evidencing bigger changes between conformers obtained in different solvents. Relative ASA differences averages were 261.39, 285.51 and 196.33 for AA, AO and OO, respectively (observed medians: 154.30, 165.10 and 103.05, respectively). P-values for relative ASA differences comparisons were in all cases << 0.001

Changes in the accessible surface area (ASA) between the maximum RMSD pairs of conformers followed the general trend shown for RMSD. We observed that both the difference in the global ASA and in the relative ASA are the lowest for the OO subgroup (averages 310.76 Å^2^ and 196.33 Å^2^) and AA (412.78 Å^2^ and 261.39 Å^2^), while differences for the AO subgroup are the highest (averages 448.66 Å^2^ and 285.51 Å^2^) (see Fig. 2a and b). The same trend was found for the global and relative ASA distributions in the control dataset (Additional file 1: Figure S1A and S1B respectively). These observations could be explained by the fact that the measurements in the OO and AA subgroups were obtained in two similar media, showing similar exposure to the solvent, while in the AO case we are observing transitions from an aqueous to a non-aqueous medium with consequent larger changes. To gain knowledge on how amino acid movements are related to these observations, we calculated the average percentages of buried amino acids for the conformers from the three subgroups. These percentages show a higher number of buried residues in non-aqueous media, as expected (48.37% for conformers in organic solvents while 44.63% in water). *P*-values for global ASA difference comparisons were in all cases < 0.001.

It is interesting to note that when the accessible surface area of all conformers for each protein were compared, we found that conformers obtained in non-aqueous media show values around the middle of the distribution of the aqueous population. This behaviour (Additional file 1: Figure S2) shows again the restricted conformations adopted in non-aqueous environments, where conformers do not tend to explore extreme conformations like their aqueous counterparts.

Finally, we have analyzed the hydrogen-bonds content in conformations from A and O conditions. We found that the average hydrogen-bonds in A is 721.18 while for O it is 847.87. Both distributions are different with a *P*-value << 0.01 (See Additional file 1: Figure S3). Again, the major differences between groups were observed for AO hydrogen-bonds differences (Additional file 1: Figure S4). These results indicate again the higher heterogeneity of A conformations compared with the O population as derived from the conformational diversity analysis (Fig. 1). The same trend is observed when radii of gyration is analyzed (Additional file 1: Figure S5).

### Conformational diversity in functionally related structures

We also studied the conformational diversity in transition subgroups (AA, AO and OO) due to changes in tunnels, cavities and active sites. Tunnels and cavities are functional structures that connect the protein surface with the active or binding site of the protein. These structures were studied on the large dataset only in those proteins having a characterized active site (see Methods). We have also characterized the presence of order-disorder transitions due to their importance in biological activity and their contribution to conformational diversity [29]. We found that tunnel length differences between conformers from subgroup OO were statistically lower than those observed for conformers from subgroups AA and AO, possibly indicating that non-aqueous conformers are more similar to each other than to the conformers obtained in aqueous solution. However, the number and length of the tunnels among the subgroups are statistically equivalent. The same behaviour was found for the number of cavities but not for their volume. Although cavities are equally distributed in the different subgroups, their flexibility (measured as the average of B-factors of all atoms of the pocket) was lower in subgroup OO, when compared with subgroups AA and AO (mean cavities flexibility 0.23, 0.25 and 0.30, respectively). We also found that cavities are larger in conformers in non-aqueous media than those found in aqueous media (mean total cavities volume 5969.58 and 5762.70 Å^3^_,_ respectively). This tendency was the same when the maximum cavity volume as well as the total volume of all cavities found in a given conformer were registered.

**Fig. 3:**
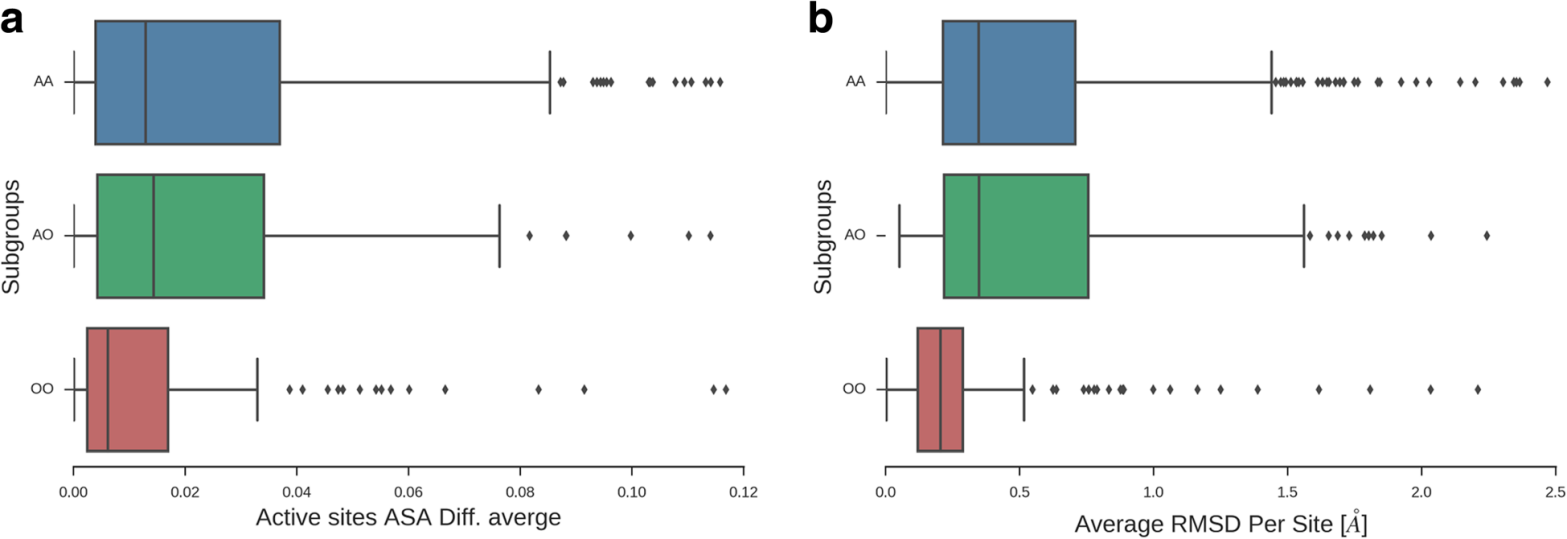
**a** Distribution of the average RMSD for residues corresponding to active site for the different transitions subgroups AA (aqueous-aqueous environments in blue), AO (from aqueous to non-aqueous environments in green) and OO (from non-aqueous to non-aqueous environments in red). Mean RMSD per site averages estimated for residues in the active site were 0.68, 0.68 and 0.32 Å for AA, AO and OO, respectively (observed medians: 0.46, 0.41 and 0.20 Å, respectively). P-values for comparisons between OO and AO and OO and AA were << 0.001 while AO and AA distributions showed no significant differences. **b** Distribution of the average ASA differences for residues corresponding to active site for the different transition subgroups AA (aqueous-aqueous environments in blue), AO (from aqueous to non-aqueous environments in green) and OO (from non-aqueous to non-aqueous environments in red). Active sites ASA average differences were 0.044, 0.043 and 0.019 for AA, AO and OO, respectively (observed medians 0.028, 0.017 and 0.01, respectively). P-values for relative ASA differences comparisons were in all cases < 0.001

Using the characterized residues needed to sustain the enzymatic activity, extracted from Catalytic Site Atlas database [30], we were able to analyze the structural differences between the conformers at their active sites. We used a total of 390 AA, 153 AO and 197 OO maximum RMSD pairs, and we found that the mean RMSD of active site residues, as well as their mean ASA for AA and AO, was significantly higher than the one observed for subgroup OO (*P*-values for RMSD comparisons between OO and AO and OO and AA were << 0.001 while AO and AA distributions showed no significant differences) (Fig. 3a and b).

Finally, following the analysis of protein flexibility [20], we quantified the differences in missing regions (see Methods) and missing residues for conformers in each subgroup. We observed the greatest differences in subgroup AO (average 0.67 and 0.03, respectively) and the lowest in subgroup OO (average 0.31 and 0.015). Moreover, the averages are the lowest among non-aqueous conformers. These results indicate that order-disorder transitions are highly affected by the presence of non-aqueous medium.

### Biological example

**Fig. 4:**
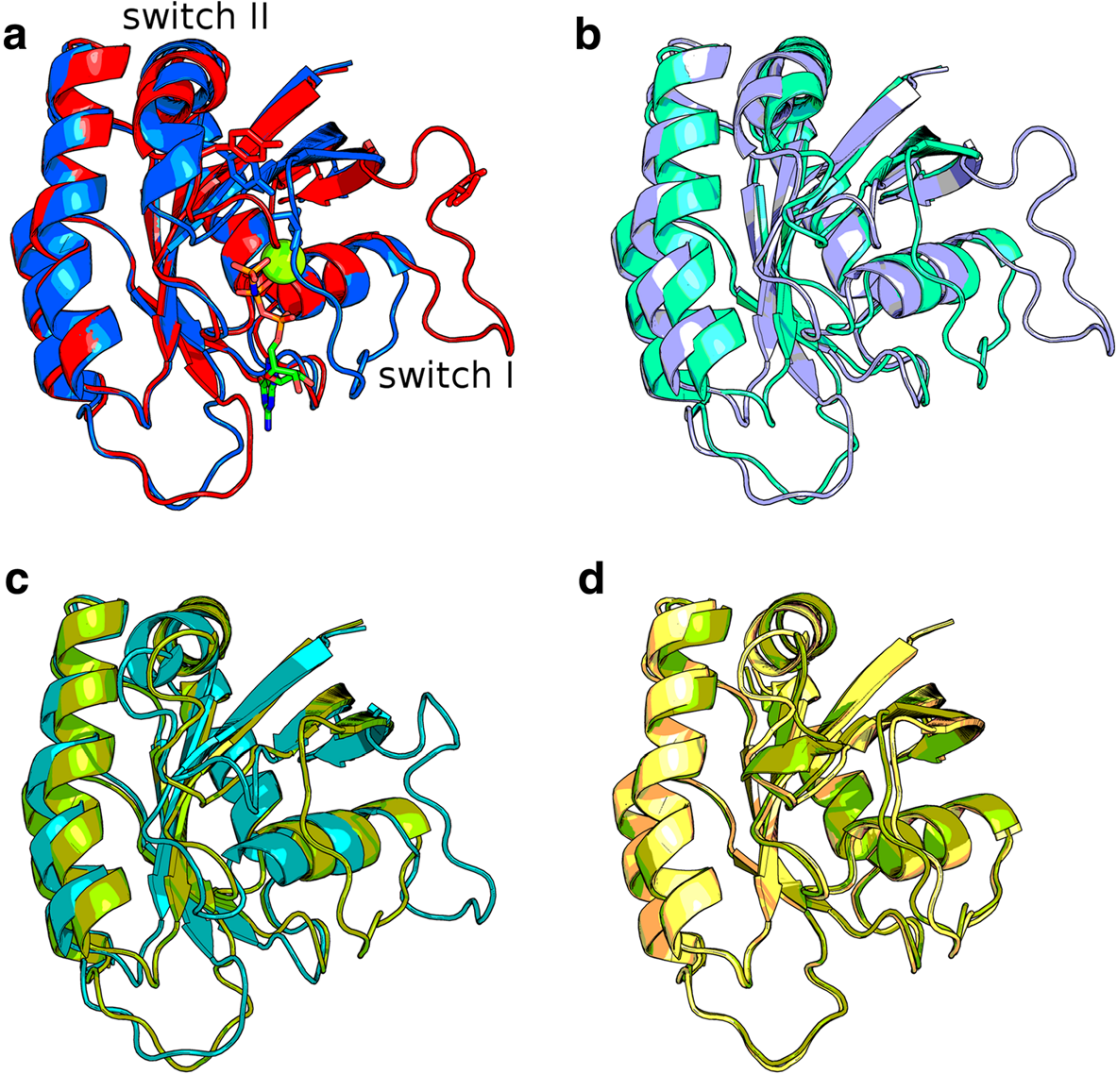
Structural representation of Ras protein conformers. **a** Cartoon representation of state 1 (red, PDB ID = 1x2d_B) and state 2 (blue, PDB ID = 1ctq_A) conformers (inactive and active respectively) of human Ras protein. In stick representation are Mg^++^ and GTP bound ligands, Thr 35 (switch I) and Tyr (switch II) essential components for Ras activity. **b** Cartoon representation of AA maximum RMSD pair, 1xd2_B (light purple) and 4dls_A (cyan) showing again the state 1 and state 2 respectively. **c** Cartoon representation of AO maximum RMSD pair, where 1p2s_A (light green) and 4nym_R (cyan) showing state 1 and state 2 respectively. **d** Cartoon representation of maximum RMSD OO pair showing 1p2s_A (light green) and 3rs5_A (light orange) showing both structures the state 2. 1p2s_A was resolved in 50% trifluoroethanol and 3rs5_A in 55% dimethylformamide

One of the major conclusions in our manuscript is that proteins in aqueous solvents show higher proportions of conformational diversity measured by maximum RMSD than those in non-aqueous solvents. An example showing this behaviour is represented by the human Ras protein. Ras protein belongs to a large superfamily of proteins known as ‘G-proteins’ with GTPasa activity [31]. When Ras is ‘switched on’ by incoming signals, it subsequently switches on other proteins, which ultimately turns on genes involved in cell growth, differentiation and survival. Ras native state is described by two main forms, state 1 and 2 or the inactive and active conformations respectively [32]. The state 1 structure is distinguished from state 2 by the loss of the interactions of Thr-35 of Ras with the phosphate of GTP. This produces a deviation of the switch I loop (residues 30–40) away from the guanine nucleotide producing an unstable and flexible conformation of the loop. Also, a Tyr residue (Tyr-64) located in another switch region, called switch II (residues 60–76), in state 1 form is too far away to exert a significant effect on the gamma-phosphate of the GTP to be hydrolyzed [32] (Fig. 4a, PDB ID 1xd2 and 1ctq).

In CoDNaS human Ras protein (Uniprot ID P01112) has 99 conformers. The AA pair showed an RMSD of 3.14 Å, the AO showed an RMSD of 3.01 Å and OO showed the minimum RMSD 0.82 Å. The same tendency was observed for ASA. These results reflect the trend already observed in Fig. 1 for the control and large datasets. In Fig. 4 we also show in panels b, c and d, the representations of AA, AO and OO pairs evidencing the conformational restrictions in the conformational diversity of OO pair. Conformational shift accompanied by order/disorder transitions in Ras protein was also described by Buhrman et al. [33]. They studied the effect of organic solvents which favored the transitions from disordered to ordered segments of Ras protein mostly in the switch II region (Fig. 4a). Also, this result in Ras protein agrees with our finding that non-aqueous conformers present lower proportions of disordered regions. Hydrophobic solvents could favour disorder to order transitions of short regions in proteins. In general, they favour H-bonding interactions between groups that are highly solvated and mobile in aqueous solutions. We have already shown that hydrogen-bonds are higher in non-aqueous conformers, a trend that is also observed in Ras conformers.

## Discussion

Our results stress the fact that proteins in a non-aqueous environment are more rigid, as many previous studies have shown [2, 34]. This finding is observed in the OO distribution of RMSD, when compared with AA and AO distributions, which are slightly above the range of the crystallographic error (~ 0.4 Å) [35]. Apparently, different structures of the same protein are almost identical in non-aqueous media independently of their bound or unbound state (average RMSD OO = 0.68 Å). However, under the kinetic trapping hypothesis, proteins in organic solvents will retain the same structure they have in aqueous media [2, 36] and in terms of our dataset the distribution of OO should show almost the same RMSD distribution as the AA distribution (Fig. 1). Considering backbone diversity, the same behaviour is observed for absolute and relative ASA (Fig. 2a and b) and the structural changes in different secondary structural elements where AO exhibits the highest variation (Table 1) when compared with AA and OO distributions. Apparently, conformers obtained using non-aqueous media shift to certain conformations avoiding the adoption of extreme conformations (complete open/close) when compared with aqueous conformers, as derived from ASA distributions (Additional file 1: Figure S2 and S3).

Nevertheless, these global structural differences do not correlate with the behaviour of tunnels, where no differences were found among the three subgroups. The number and length of tunnels do not show differences between A and O type conformers. However, it is interesting to note that our results show that cavity volumes are larger in O conformers than in A conformers. Cavities normally found in proteins are generally associated with active sites of enzymes or binding sites of transporter proteins [37]. It has been shown that while non-polar cavities become larger, they are stabilized by a cluster of mutually interacting water molecules [38]. However, proteins in organic solvents could increase their cavity volume due to the entrance of organic solvent molecules, without further changes in the overall topology of the protein [39], a finding that could explain our results.

## Conclusions

Our findings suggest some discrepancies with the predictions made by the kinetic trapping hypothesis. We found that conformers in non-aqueous media have a lot less conformational diversity than those in aqueous media; conformers in non-aqueous media also have larger cavities, fewer solvent exposed surfaces and fewer disordered regions. As protein dynamism is a key feature to sustain biological function [40], as well as to ensure the preservation and dynamic behaviour of cavities and pockets [41] and order/disorder transitions [27], the specific features described above for conformers in organic media could contribute to explain their lower biological activity.

## Methods

### Dataset building

The information about solvent concentration and experimental procedures applied to protein crystallization is not always available from the PDB files (i. e. incomplete or absent information). To solve this problem, we built a consensus list of organic solvents and non-aqueous crystallization media which are commonly used in the crystallization process; to do this, we referred to crystallographic manuals and research articles. Then, we used this list to search crystal structures (without mutations and resolution < 4 Å) from the database of Conformational Diversity in the Native State of proteins (CoDNaS) (a conformational diversity database, based on a collection of redundant structures for the same protein, linked with physicochemical and biological information) [26]. The presence of these organic molecules in the crystal, indicated in the HETATOM field of the PDB files, was used for distinguishing the aqueous from the non-aqueous environment structures, and for building the “large” dataset. The large dataset then contains 1737 proteins with 3474 conformers. We also considered another dataset resulting from the web scrapping method and hand curation for the collection of structures related to soaking and co-crystallization methods in organic solvents, which contained 33 proteins and 2755 structures. In this case, the structures were collected using the web scrapping method, in which bibliographic databases were explored to gather research articles related with soaking and co-crystallization methods in organic solvents and/or non-aqueous media. Using the text mining method, all the articles found were analyzed and related to a PDB structure. The structures obtained were linked with their respectively CoDNaS entries in order to get the conformers for each protein. This last dataset was considered as a “control” one and all its tendencies were contrasted with those in “large” ones. Pairs of conformers were explored for the presence of bound ligands, in order to obtain bound-bound and unbound-unbound pairs of conformers to avoid bias in the analysis of conformational diversity. Presence of bound ligands was evaluated using BioLiP database [42].

Both datasets were presented and analyzed as having three subgroups of pairs of conformers: those in which both conformers contained any of the common organic solvents and/or non-aqueous media used in protein structure estimations in our list (see Additional file 1: Table S1) or were structures obtained from research articles related with co-crystallization and soaking in organic solvents (OO); those in which only one of them had the organic molecules in its crystal (AO); and those in which no organic solvent was found (AA). In each set, we only considered the highest C-alpha Root Mean Square Deviation (RMSD) between the corresponding conformers for a given protein. Therefore, we obtained three subgroups for the large dataset (AA, AO and OO with 9680, 1737, 2062 pairs of conformers, respectively) and three subgroups for the control dataset (AA, AO and OO with 33, 31, 25 pairs of conformers, respectively).

### Structural characterization

To estimate the structural dissimilarity between conformers, we used the C-alpha RMSD, which was calculated using MAMMOTH [43]. The accessible surface area (ASA) is the surface area of a biomolecule that is accessible to a solvent. ASA calculations for each conformer were obtained using NACCESS (S. Hubbard and J. Thornton. 1993. NACCESS, Computer Program. Department of Biochemistry Molecular Biology, University College London). Global ASA corresponds to the sum of absolute ASA values of each residue and relative ASA is calculated for each amino acid in the protein by expressing the various residue accessible surfaces summed as a percentage of that observed in a ALA-X-ALA tripeptide.

To obtain a measurement of the amino acid movements, we have calculated the amount of amino acids buried (ASAs lower than 25% were considered buried, and ASAs over 25% were considered exposed) for the three populations. All the data was processed using our own scripts coded in Python.

To explore the transitions between the different secondary structures, we defined the secondary structure for each conformer using DSSP [44]. The C-alpha and residue atoms RMSD per position was calculated using ProFit (Martin, A. C. R. and Porter, C. T. http://www.bioinf.org.uk/software/profit/). Disorder was assumed as represented by missing electron density coordinates in a structure determined by X-Ray diffraction [45]. To define intrinsically disordered regions (IDRs) we only considered those segments with five or more consecutive missing residues which were not in the amino or carboxyl terminal ends of the protein sequence (the first and last 20 residues of the chain were excluded). Fold class and superfamily were studied using CATH database [46]. As control and large dataset showed the same trend in terms of backbone RMSD, these structural analyses were performed only in the large dataset.

All data obtained were retrieved and processed using home-made scripts coded in Python.

### Radii of gyration and H-bonds

Radii of gyration for all PDB structures were estimated using the MMTSB tools (http://blue11.bch.msu.edu/mmtsb/Main_Page). For the calculation of the number of hydrogens bonds we used HBPLUS [47]. Comparisons between conformers were made using our own Python scripts.

### Tunnels and cavities calculation

The number of cavities and tunnels, as well as their properties, were estimated for all conformers using Fpocket [48] and MOLE [49]. All data obtained were retrieved and processed using our own scripts coded in Python.

### Statistical tests

Dataset distributions were assumed to be continuous and not parametric, which was confirmed by D’Agostino and Pearson’s normal test. Comparisons within groups were made by Kolmogorov-Smirnov test, as appropriate. One-way ANOVA was used for multigroup comparisons. A *P*-value < 0.05 was taken to indicate statistical significance.

## Additional file

Additional file 1: Supporting online material. PDF document with supplementary figures (Fig. S1–S5) and table S1. (PDF 266 kb)

## Abbreviations

A: Aqueous
ASA: Accessible Surface Area
O: Non-aqueous
PDB: Protein Data Bank
RMSD: C-alpha Root Mean Square Deviation
