# Supplementary material for: Large scale analysis of protein conformational transitions from aqueous to non-aqueous media
Source: BMC Bioinformatics. 2018 Jan 30;19:27. doi: 10.1186/s12859-018-2044-2 (PMC5791380; doi:10.1186/s12859-018-2044-2)
Supplement: Additional file 1: — Supporting online material. PDF document with supplementary figures (Fig. S1–S5) and table S1. (PDF 266 kb) [file 12859_2018_2044_MOESM1_ESM.pdf]

Large scale analysis of protein conformational transitions between aqueous to non-aqueous media

Ana Julia Velez Rueda[1], Alexander Miguel Monzon[1], Sebastián M. Ardanaz[2], Luis E. Iglesias[2] and Gustavo Parisi[1, *].

[1]Departamento de Ciencia y Tecnología, Universidad Nacional de Quilmes, Roque Sáenz Peña 352 - Bernal, B1876BXD, Provincia de Buenos Aires, Argentina.

[2]Laboratory of Biocatalysis and Biotransformation, Departamento de Ciencia y Tecnología, Universidad Nacional de Quilmes, Roque Sáenz Peña 352 - Bernal, B1876BXD, Provincia de Buenos Aires, Argentina.

*To whom correspondence should be addressed: E-mail, gusparisi@gmail.com.

Supplementary Information

A B

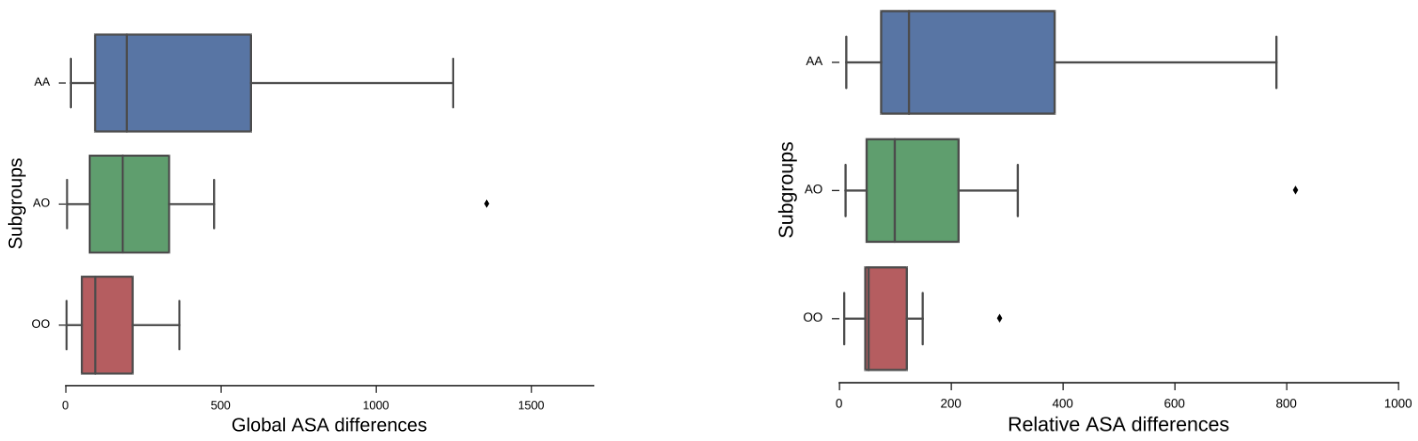

Figure S1. A. Differences in the global ASA for the different subgroups AA(blue), AO(green) and OO (red) for the control dataset. AO subgroup show the maximum differences evidencing bigger changes between conformers obtained in different medium. Global ASA differences averages were 424.11, 328.12 and 140.62 for AA, AO and OO respectively (observed medians:196.33, 183.05 and 94.81 respectively). P-values < 0.05. S1B.The relative ASA differences for the control dataset shows the same behavior than the global ASA differences (1A).

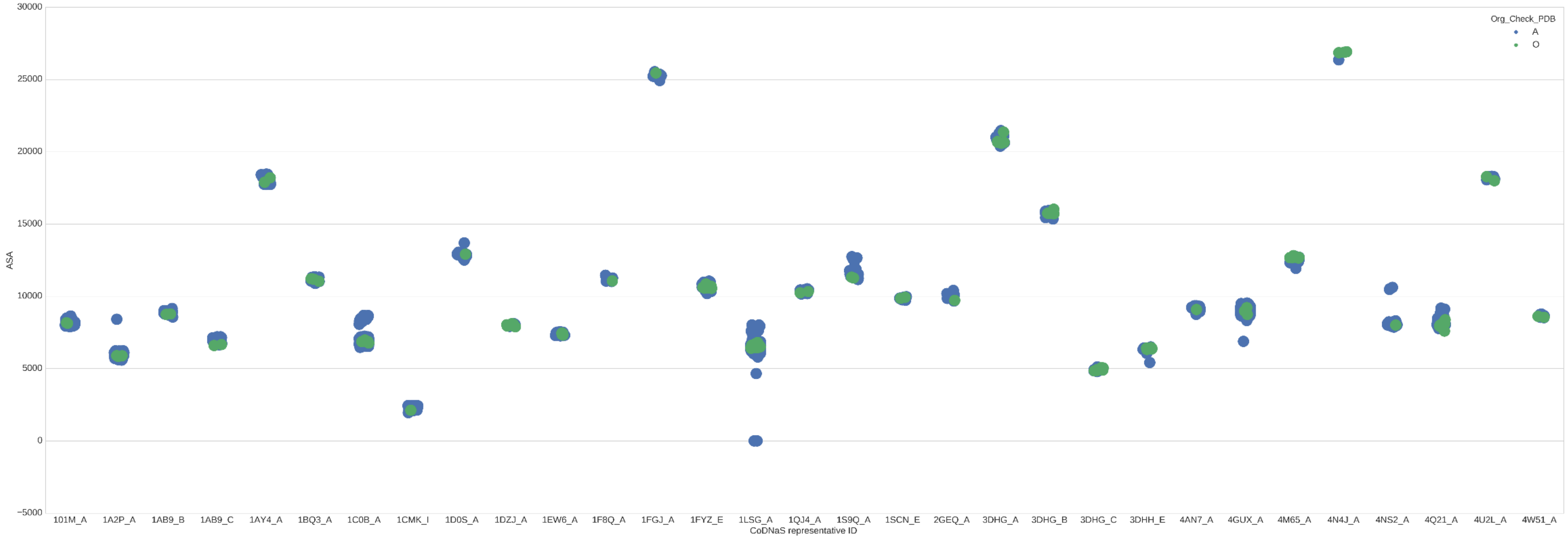

Figure S2. Global ASA distributions taking into account all the conformer pairs. Distributions of accessible surface area of a representative pool of proteins from the "control" dataset measured in ASA (Å^3) for the A and O conformers. The accessible surface area for aqueous (A) conformers shown in blue and the accessible surface area for non-aqueous conformers (O) are shown in green.

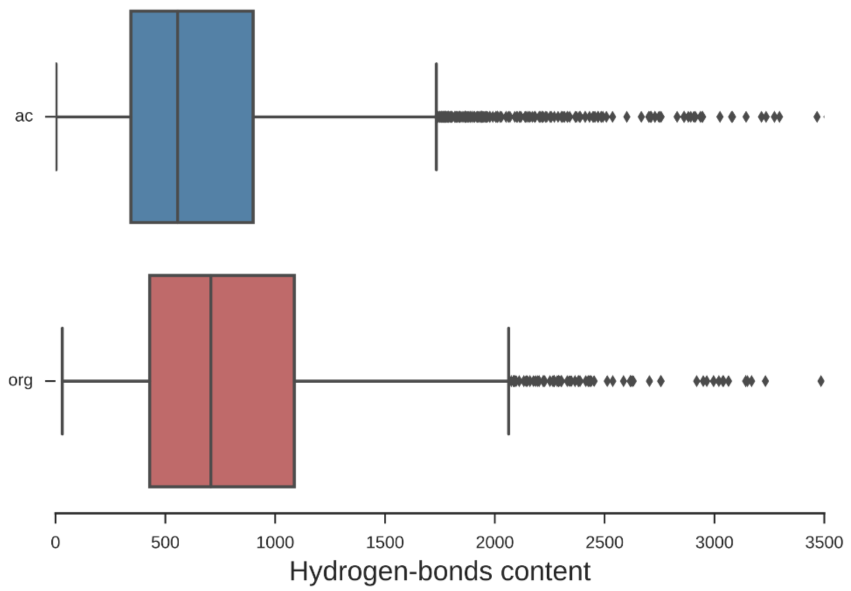

Figure S3. Hydrogen-bonds distributions for the aqueous and non-aqueous protein conformers in large dataset. For aqueous conformers (shown in blue) hydrogen-bonds average is 721.18 while for non-aqueous conformers is 847.87.

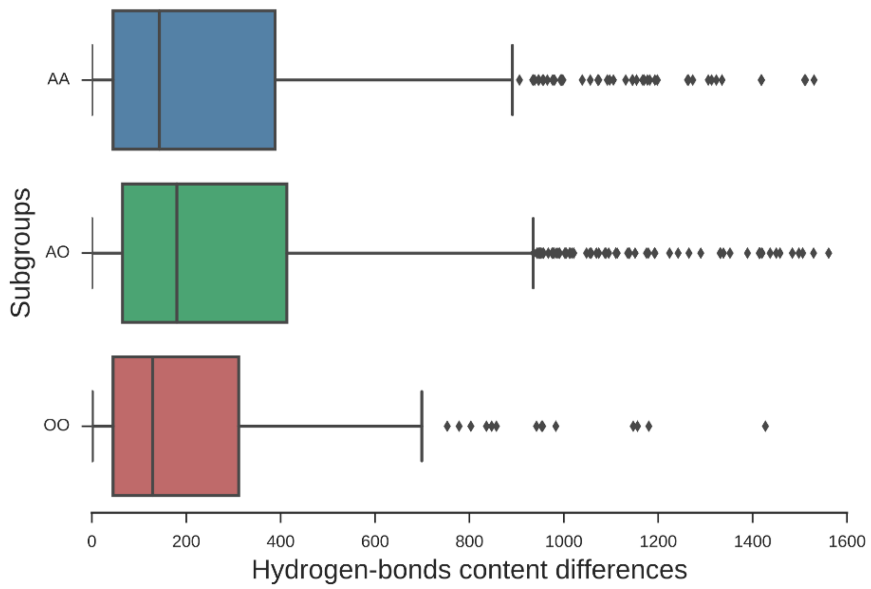

Figure S4. Distributions of the difference in the hydrogen-bonds count for each subgroup in the large dataset. Differences average for AA (shown in blue) was 315.87 (median 143), for the AO subgroup (shown in green) the mean value was 312.71 (median 180), and for the OO (shown in red) the mean value was 246.57 (median 129). Statistical difference was P-value << 0.01 while comparing AA vs AO and OO vs AO. No statistical difference was found between AA and OO distributions.

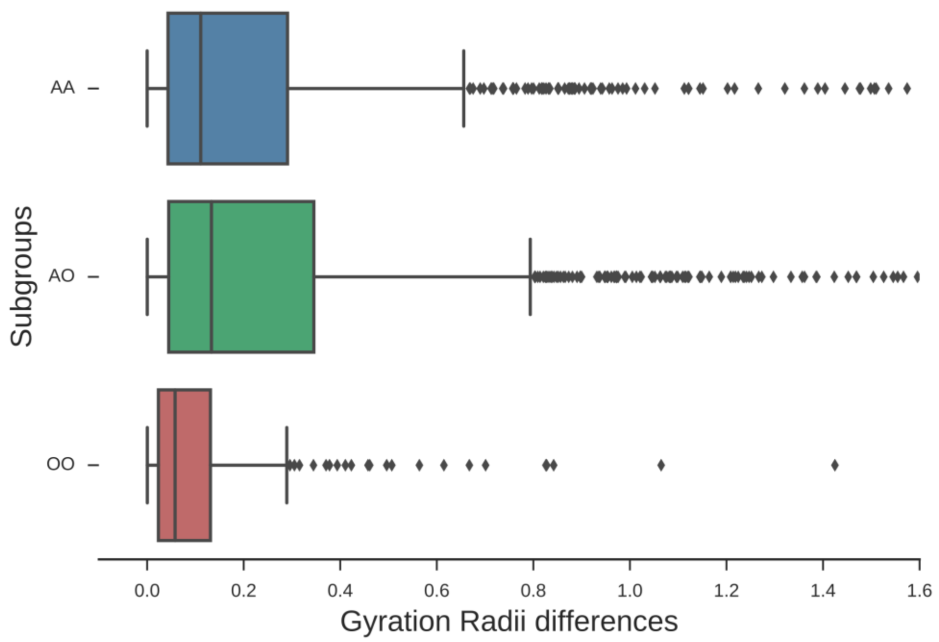

Figure S5. Radius of gyration difference distributions for each subgroups of the large dataset. Mean difference gyration radii was 0.275 for AA (shown in blue), 0.392 for AO (shown in green) and 0.146 for OO (shown in red). P-value<< 0.01 for all the comparisons.

PDB Code	Organic solvents or/and organic molecules present in protein structure estimation
3GR	glyceraldehyde
4PB	butanamide
8CL	chlorobenzene
ACN	acetone
ANF	anthrone
ARF	formamide
BML	4-bromophenol
BU1	butane-1,4-diol
BXO	butyraldoxime
BZF	benzofuran
CCN	acetonitrile
CXL	cyclohexanol
CYH	cyclohexanone
D12	dodecane
DIO	1,4-dioxane
DMF	N,N-dimethylformamide
DMS	dimetyl sulfoxide
EOH	ethanol
ETF	trifluoroethanol
ETI	iodoethane
GOL	glycerol
HEX	hexane
HEZ	hexane-1,6-diol
HP6	heptane
HY1	phenylacetaldehyde
IPA	isopropanol
IPH	phenol
MBN	toluene
MBR	tribromomethane
MCH	chloroform
MEE	methanethiol
MOH	methanol
NBU	butane

NBZ	nitrobenzene
NEH	ethylamine
OCT	octane
PHZ	phenylhydrazine
POL	propanol
PTD	pentanedial
PYJ	ethylbenzene
SCN	thiocyanate
UNA	undecanal

Table S1. Solvents commonly used and organic molecules present in protein structure estimation. PDB Ligand ID and names.
